# EXP-Crowd: A Gamified Crowdsourcing Framework for Explainability

**DOI:** 10.3389/frai.2022.826499

**Published:** 2022-04-22

**Authors:** Andrea Tocchetti, Lorenzo Corti, Marco Brambilla, Irene Celino

**Affiliations:** ^1^Dipartimento di Elettronica, Informazione e Bioingegneria, Politecnico di Milano, Milan, Italy; ^2^Cefriel, Milan, Italy

**Keywords:** explainability, crowdsourcing, gamification, game with a purpose, Explainable AI

## Abstract

The spread of AI and black-box machine learning models made it necessary to explain their behavior. Consequently, the research field of Explainable AI was born. The main objective of an Explainable AI system is to be understood by a human as the final beneficiary of the model. In our research, we frame the explainability problem from the crowds point of view and engage both users and AI researchers through a gamified crowdsourcing framework. We research whether it's possible to improve the crowds understanding of black-box models and the quality of the crowdsourced content by engaging users in a set of gamified activities through a gamified crowdsourcing framework named EXP-Crowd. While users engage in such activities, AI researchers organize and share AI- and explainability-related knowledge to educate users. We present the preliminary design of a game with a purpose (G.W.A.P.) to collect features describing real-world entities which can be used for explainability purposes. Future works will concretise and improve the current design of the framework to cover specific explainability-related needs.

## 1. Introduction

Over the last decades, the development of new Artificial Intelligence (AI) technologies brought forth the necessity of improving their understandability. In Explainable AI (XAI), most researchers develop algorithms to either explain models or improve their intrinsic explainability. The main problem associated with the understandability of an AI system is the gap between the explanation and the level of understanding of non-expert people. Such a gap is mainly influenced by the shape of the explanation (i.e., textual, visual, low-level details, etc.), its complexity, the persons level of knowledge, and many other factors associated with both the model and human side. In particular, while sometimes it is possible to re-shape the explanation to improve its understandability for non-experts, it is challenging to leverage people's knowledge as they are usually engaged in validation and data collection activities.

Alongside the development of AI systems, the need for training and labeled data has grown as well. Therefore, resorting to crowdsourcing has become essential to collect knowledge at scale. As such processes can sometimes be tedious and repetitive, researchers developed strategies to improve their design and effectiveness. In particular, Von Ahn (2006) proposed a *human computation paradigm* influencing the design of crowdsourcing activities, the so-called “Games With A Purpose” (G.W.A.P.). Such a paradigm enhances crowdsourcing endeavors through Gamification (Hamari, 2019), making them more entertaining for the people to partake.

Our research longs for envisioning an open gamified crowdsourcing framework with the final aim of (1) improving the capability of the crowd to understand black-box AI models explanations, (2) improving the quality of the explanations provided by a black-box model by engaging the crowd to provide helpful content to AI practitioners, and (3) evaluating whether providing structured AI-related knowledge and engaging the crowd in explainability-related activities is an efficient way to achieve these objectives. As a first use case, our research covers image classification models. We explore user engagement, gamification, and knowledge collection and structuring to answer our research questions. Ultimately, we strive to create an open community through which users learn to understand the behavior of black-box models, therefore, providing value for both the developers and themselves.

The rest of this article is structured as follows. Section 2 provides an overview of explainability, crowdsourcing, and gamification. Section 3 outlines the preliminary framework design we envision, including a use case of a gamified activity for data collection and structuring and some use cases. Section 4 discusses the main advantages and limitations of the framework and explains how to overcome such restraints. A discussion on the gamified activity is also provided. Finally, Section 5 summarizes the critical contributions featured within this article and discusses the following research steps.

## 2. Related Works and Background

### 2.1. Explainability

One of the most well-known Artificial Intelligence (AI) branches is Machine Learning (ML). In ML, algorithms train models to perform predictions, classifications, groupings, and other tasks by learning from data. The development of Deep Learning (DL) and Deep Neural Networks (DNN) increased Machine Learning models' accuracy and performance at the expense of their interpretability. Indeed, most DNNs are referred to as “black-box” (or opaque) models. The input and output of a black-box model are known, while it is complex to understand its internal logic. They are opposed to “white-box” models, in which the internal logic is either known or easily understandable.

As of today, there is no unique definition of model explainability (Vilone and Longo, 2020). Despite the ongoing research efforts to define the fundamentals of an explainable AI system, most definitions are either domain- or problem-specific and are usually used interchangeably across different research fields (Guidotti et al., 2018). In the definitions provided by Barredo Arrieta et al. (2020), the notion of “human understandability” is the most important concept associated with Explainable AI. At the same time, other scholars consider different concepts depending on their research focus, like transparency (Belle and Papantonis, 2020) and explainability (Guidotti et al., 2018, Hu et al., 2021). In their definition of Explainable AI, (Barredo Arrieta et al., 2020) highlight that the understandability of an explanation is influenced by the ones to whom it is provided, i.e., *the audience*. In particular, depending on the person's knowledge about AI and ML, an explanation can be shaped differently. For example, an AI expert would probably prefer a detailed model description. On the other side, an inexperienced user would favor a small set of examples describing the system's behavior. Moreover, the authors state that an AI must generate an explanation “*clear or easy to understand,”* even though the concept of being easy to understand is not the same for everyone.

Regardless of the variety of explainability-related definitions provided in the literature, the researchers' community agreed that the main objective of an Explainable AI system is to be understood by a human as the final beneficiary of the model. Despite such an objective, XAI studies mainly approach the problem from a model-centric perspective rather than a user-centric one, overshadowing the level of users' understanding of the model. In particular, end-users and experts are frequently engaged in the later validation stages to evaluate the level of understandability of the model either directly or through simulated user experiments (Ribeiro et al., 2016; Lundberg and Lee, 2017).

### 2.2. Crowdsourcing and Gamification

Artificial Intelligence methods—especially Deep Learning approaches—require a large amount of high-quality data, whose collection is demanding and challenging. The widespread use of the internet allows researchers to engage virtually unlimited people to cover their data needs. Indeed, crowdsourcing has become common and encompasses academic studies and private companies' interests. Crowdsourcing can be defined as a participative online activity in which a group of individuals with varying features is engaged in undertaking a task as part of a process mutually benefiting participants and crowdsourcers (Estellés-Arolas and de Guevara, 2012). This methodology's advantages include lower costs, greater speed, and a higher degree of diversity by engaging a large and heterogeneous pool of people. This open-source practice either allows the collection of a wide variety of data, including peoples ideas and preferences (Balayn et al., 2021b), or the accomplishment of a task (e.g., labeling a large number of images) (Mishra and Rzeszotarski, 2021).

Sometimes, crowdsourcing is enhanced with gamification (Hamari, 2019) to make such a process more engaging, drive users' behaviors, and structure the collected data. Gamification uses people's motivations to achieve such objectives. Ryan and Deci (2000) accurately describe the influence of motivations on human decisions, mainly distinguishing between *intrinsic* and *extrinsic* motivations. Their definitions can be summarized as “*the motivation to perform a behavior or engage in an activity for our own sake rather than the desire for some external reward”* and “*the motivation to perform a behavior or engage in an activity due to a separable outcome”* (Lee et al., 2016), respectively. Following such a dichotomy, gamified approaches can be organized based on the kind of motivation they leverage. For example, pointification, leaderboards, etc. affects extrinsic motivation while receiving feedback (Hamari and Koivisto, 2013) and learning (Cerasoli et al., 2014) influence the intrinsic one. Moreover, an extrinsic-oriented design results in a good initial level of engagement, while it is necessary to apply an intrinsic-oriented design to achieve a long-lasting engagement (Rapp, 2015).

Gamification and G.W.A.P. have also been widely applied in computer science. Lu et al. (2021) developed a *Peek-a-boom*-based XAI evaluation, demonstrating the presence of differences between crowd-based and automatic assessment. Balayn et al. (2021a) developed *FindItOut*, a game with a purpose based on the *GuessWho* game with the final aim of collecting and organizing knowledge for researchers and AI practitioners. Speer et al. (2009) presented a gamified interface to acquire common sense knowledge through a *20 Questions* game which motivates contributions and improves the throughput of new knowledge. Other than contributing to data collection, it has also been demonstrated that Gamification can be effective in education and learning (Buckley and Doyle, 2016, Welbers et al., 2019). In particular, leveraging intrinsic motivation through feedback cycles is an effective way to enhance learning (Lee and Hammer, 2011).

## 3. Methods

**Figure 1:**
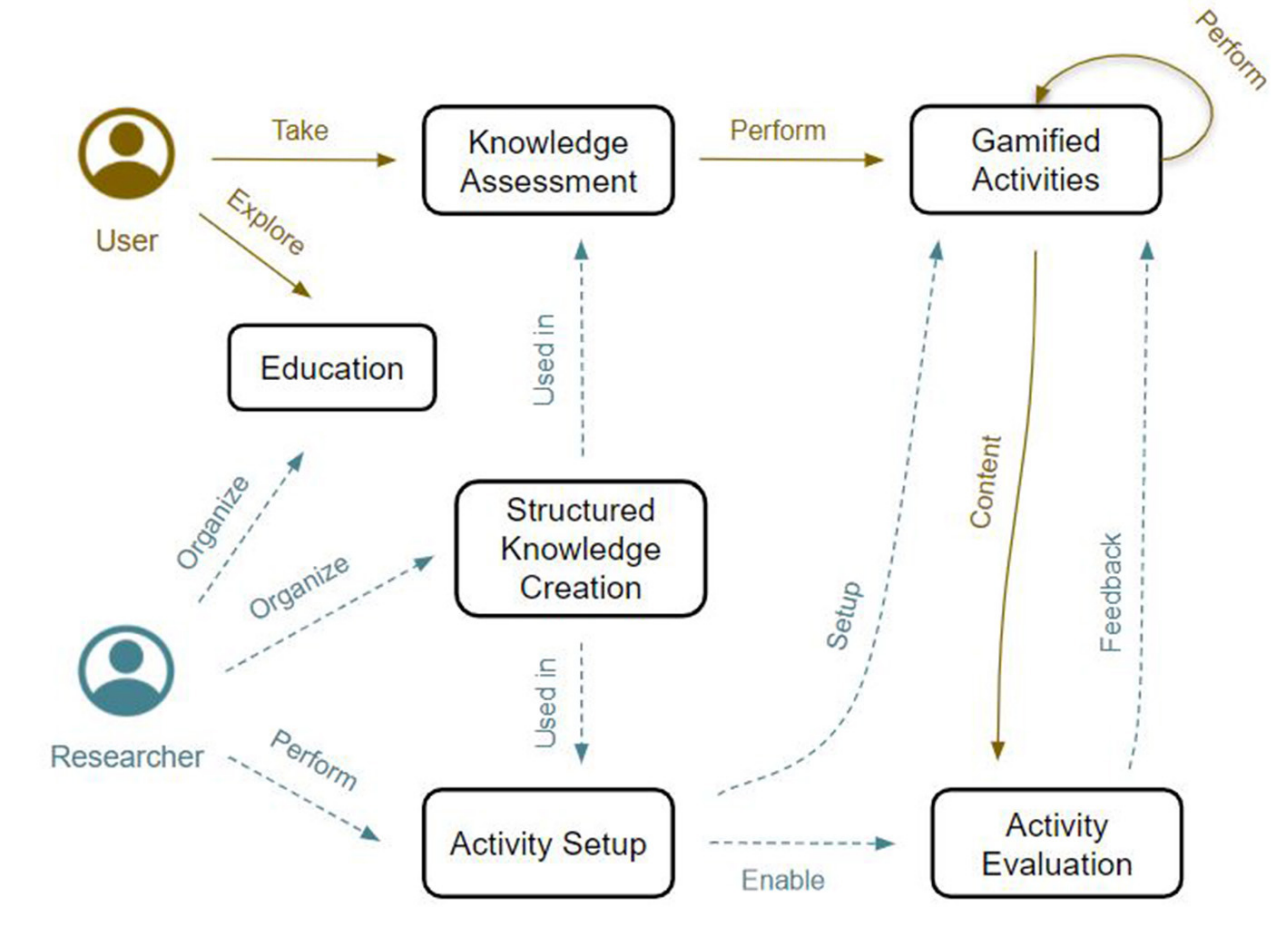
Interaction flows of researchers (dashed cyan arrows) and users (orange plain arrows) with the activities devised within our framework, as described in Section 3. Researchers organize users' knowledge and set up activities to collect data. As users engage with such activities, they provide Content to researchers. In turn, researchers give the user feedback about the activity they performed. Such feedback aims to improve users' understanding of the activity itself, the knowledge and the context provided within it.

The main actors engaged within our explainability-oriented crowdsourcing framework fall into two categories: *users*, who get involved by playing gamified activities, and *AI practitioners/researchers*, who set up games and share knowledge about AI, ML, and explainability, since they exhibit a high level of understanding of these fields. Figure 1 provides a simple overview of the interaction flow proposed within the framework.

The following sections describe each part of the framework and provide some use cases to clarify their structure. These will be mainly associated with the researcher side since most of the activities described for the user side are simple. We use a persona named “Bill” to represent our researcher. We will illustrate how he explores and interacts with the final implementation of our framework, i.e., a web-based platform.

### 3.1. Knowledge Assessment

As one of the main objectives of our framework is to improve the capability of the crowd to understand black-box models' explanations, educating users on AI-related topics is essential. Therefore, the first step is an assessment questionnaire through which their knowledge about AI and explainability will be assessed. Users will be asked to answer a series of multiple-choice questions. Depending on their results, they will be assigned a category representing their level of expertise. Users can improve their category by engaging with the proposed activities and enhancing their skills.

The research community will be requested to build a collection of multiple-choice questions employed in the assessment questionnaire and within the activities. Each question is made of (1) a set of texts through which the question is asked, (2) a set of correct answers, (3) the explanation associated with each correct answer, (4) a set of incorrect answers, (5) a difficulty score, and (6) a category. Questions must receive the approval of the community to guarantee the quality of the content provided. Therefore, each question must undergo a period of evaluation in which the community members can improve them by suggesting updates and proposing new answers and explanations. After this period, it is approved if the question received enough positive evaluations. Approved questions will be openly available to the whole research community as researchers may want to re-assess the users' knowledge as they engage with one of their activities. After a question is approved, researchers can still improve it by providing new content for elements (1–4).

***Use Case—Researcher****. Bill is a researcher who needs data about real-world entities for his research. When exploring the platform, Bill discovers a picture-based activity that would fit his needs. Even though he would like to set it up immediately, he also wants to evaluate the knowledge of the users who will perform his activity beforehand. Therefore, he explores the section dedicated to creating multiple-choice questions about AI, looking for questions that fit the context of his research. Unable to find questions that suit his needs, he submits new questions. A few days after his submission, he noticed that the researcher community proposed some improvements for the questions (e.g. by providing new answers). Bill approves a few of them. After a few more days, the question is approved*.

### 3.2. Education

Following the initial assessment, users will be schooled while engaging with the framework. In particular, knowledge will be provided in different shapes. The following list describes how knowledge about AI, ML, and explainability will be provided to users.

- **Questionnaire:** Researchers may set up a small quiz before their activity made of an arbitrary number of approved questions. For each question, they choose its text, the list of answers, and the explanation of the correct answer. Such a quiz would provide knowledge to users through the questions' explanations while allowing researchers to evaluate the level of education of the people playing the activity.
- **Knowledge Sharing:** Researchers can summarize, organize, and share knowledge by setting up tailored content for the users to read and study (i.e., the summary of a paper, the outline of the knowledge related to a specific AI topic, etc.). Each publication is made of a title, the topic it discusses, a brief description of the content, and the content itself. Researchers can also share scientific articles for the users to read. Only minimal information will be collected and shared like title, authors, and DOI. Users should access such articles by themselves.
- **Debating:** Researchers and users can discuss subjects of interest in a forum-like fashion. We argue that debating with knowledgeable people would improve the users' knowledge.

***Use Case—Researcher****. Bill would like users to understand how machine learning models learn so that users performing his activity can provide better inputs. Therefore, he collects knowledge from scientific documents, summarizes it, and shares it in the “Education” section. Bill achieves his first publication entitled “Understanding the way ML models learn from pictures: A simplified overview.” He also provides a custom picture and a few references to the articles he used to write it within the publication. Bill reads an exciting article about his research topic a few days later. As it may improve the users' knowledge even further, he shares it by providing the necessary information*.

### 3.3. Gamification

Gamified activities are the core elements of our framework. The following sections discuss the steps a researcher must accomplish to set up and evaluate the outcomes of an activity.

**Activity Setup**. AI practitioners can pick between pre-defined activities and set up the necessary content depending on their needs. These activities range between data collection, explainability evaluation, etc.

Setting up an activity involves a set of passages, depending on the activity. In general, all setup processes share a questionnaire setup step, a context setup step, and an activity setup step. In the first setup step, researchers decide whether to include a Questionnaire (as described in “Education”) and potentially organize its questions. In the second step, the researcher is asked to set up the content provided to the users to understand the context of their research, relevant concepts to know while carrying out the activity, etc. Finally, they have to provide all the necessary material to set up the actual activity. Practitioners can include additional control questions to the questionnaire and the actual activity to keep track of the user's level of attention. Practitioners can also select an advised knowledge level to provide an overview of the complexity of the concepts presented within the activity.

***Use Case—Researcher****. Bill is finally ready to set up his first activity. In the questionnaire setup step, he picks the questions (including the ones he got approved before), their answers and their explanations. In the context setup step, he provides the context of its research, describing what it consists of. Bill also provides some of the content from the knowledge summary he shared for those who didn't read it. As the last step, he gives the pictures, labels, and necessary content for the picture-based activity*.

**Activity Evaluation**. Users are only asked to play gamified activities while researchers perform many different tasks regarding the gamified activities. In particular, they can visualize relevant statistics about the users that partook in the activity they set up, including the answers to the questionnaire (if present), the outcome of the activity, whether the user successfully answered the questions, the knowledge level of the users, etc. The role of the researcher in this final step is to evaluate the users and potentially provide feedback. They have to identify those users who stood out, like those who answered correctly to a high number of questions (compared to their level of knowledge), those who carried out a high-quality activity, etc. These users will be consequently awarded. In particular, these users will be awarded status-based awards that will make them distinguished community members.

***Use Case—Researcher****. After a few weeks from publishing his activity, Bill overviews its outcomes. He notices that most users performed well while others outlined the pictures improperly. He picks the users who performed outstandingly and awards them. These users will be notified, and the award will be exhibited on their profiles. As one of the users answered most of the questions incorrectly and provided poor activity outcomes, Bill wrote them some advice on how to carry out the activity, also explaining some details related to how ML is applied in his research*.

### 3.4. Gamified Activity: A Case Study

Finally, we describe a case study on image classification and understanding, which we use as proof of concept of a gamified activity to collect data to be employed in the field of explainable AI.

When addressing the explainability of image classification models, the crowd is usually engaged to highlight, label, and detail pictures. We assert that the outcome of such a task strictly depends on the images supplied, i.e., a person describing different pictures of the same entity may provide different details. In particular, we argue that the description of a subject, provided its picture, may be limited to or by the features displayed. Therefore, we claim it would be possible to improve the collected features by unbinding the images from the process since the person won't be limited by the representation of the entity they describe. In particular, we would like to answer to the following questions

- **(Q1) Is the picture displayed to the annotator causing bias when asked to describe the entity in the image?**
- **(Q2) Are we able to collect more features with respect to the standard annotation methods?**

Therefore, we design and evaluate the effectiveness of a Game With A Purpose (G.W.A.P.) to collect knowledge in terms of relevant features and descriptions of the analyzed content. Such features are organized in three categories, namely “abstract” (identified with “A,”) “not represented in the picture” (identified with “NR,”) and “represented in the picture” (identified with “R.”) “R” features and “NR” features both represents “concrete features.”

Inspired by Ahn et al. (2006), we designed a gamified activity where a pair of people play a guessing game. The game involves the following steps.

**Figure 2:**
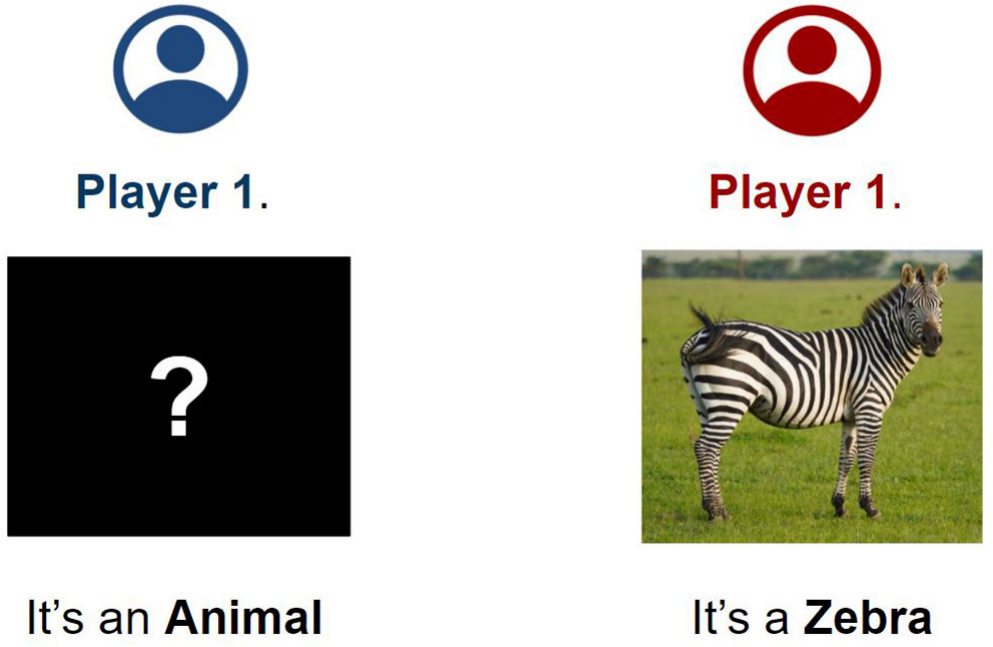
The setup step of the gamified activity. *Player 1* is provided with the category of the entity they have to guess (in this case, they have to guess an animal). *Player 2* is supplied with a picture of the entity and its name (in this case, they are provided with the picture of a zebra).

**Figure 3:**
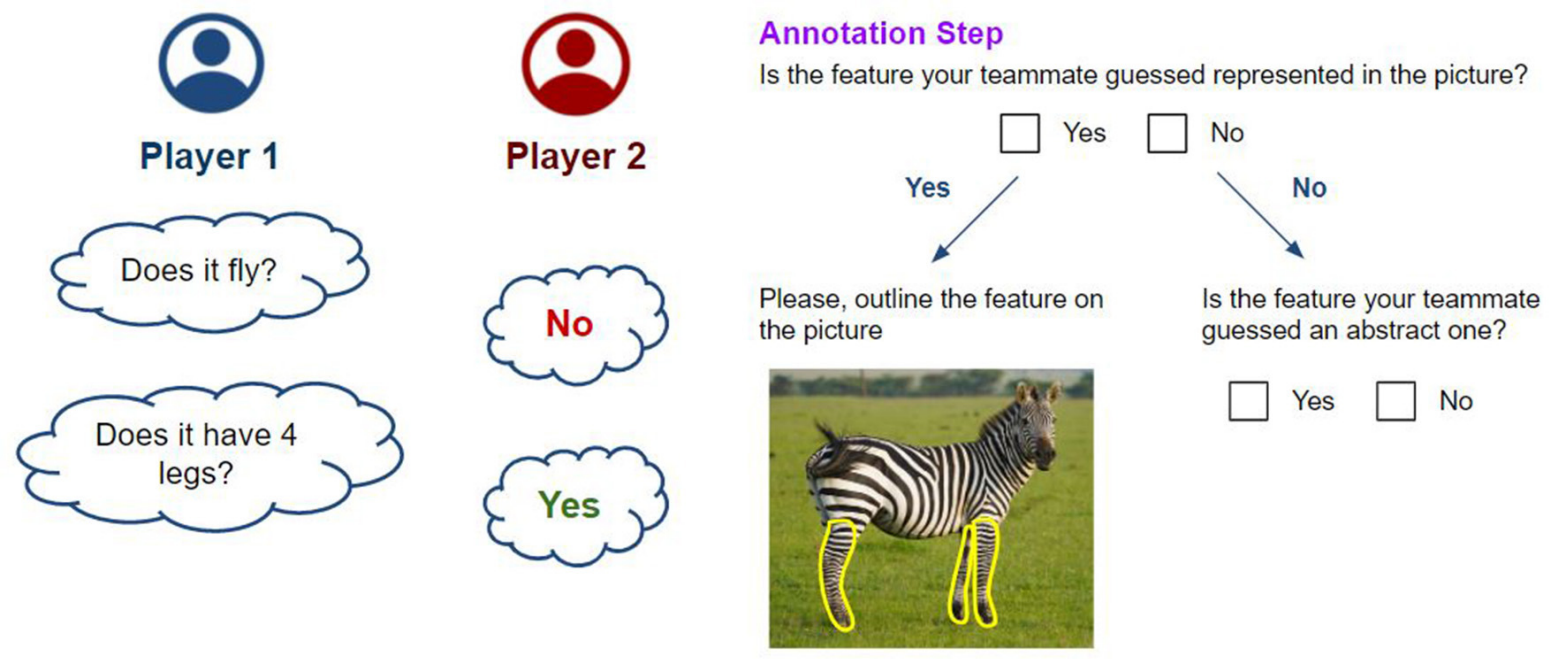
On the left, the Basic Turn of the gamified activity is displayed. *Player 1* asks yes or no questions about the entity. *Player 2* answers such questions. On the right, the Annotation Step is summarized. *Player 2* is asked to complete a series of simple tasks to identify the guessed feature by answering questions and potentially annotating the picture.

- **Initial Setup** step (Figure 2). *Player 1* is provided with the entity category they have to guess. *Player 2* is shown the picture of the entity and its name.
- **Basic Turn** (Figure 3, on the left). *Player 1* asks closed questions about the features of the entity to guess. *Player 2* answers the questions. *Player 1* may either ask questions freely or fill in predefined question templates (i.e., “Does it have ...?,” “Does it ...?,” etc.). If the answer is affirmative, *Player 2* is asked to carry out the **Annotation Step**.
- **Annotation Step** (Figure 3, on the right). Whenever the answer to a question is affirmative, *Player 2* is asked to perform a series of simple tasks to identify the guessed feature in the picture they were provided with, if possible. First, they are asked whether the element is displayed in the image. If so, they are requested to outline them in the picture. Otherwise, they are asked whether the feature is an abstract one.
- **Hint Step**. If *Player 1* guessed no features of the unknown entity in the last few questions, *Player 2* provides a bit of advice by providing a feature of the entity to *Player 1*. If possible, *Player 2* should provide a feature that *Player 1* already tried to guess. Therefore, *Player 1* will be able to proceed with the activity. *Player 2* is still required to carry out the **Annotation Step** for the hinted feature as it will still be considered in the final set of features.
- **Game Conclusion**. Finally, after *Player 1* has collected enough clues on the entity they are trying to guess, they can provide their final answer. If the answer is correct, the game is over; otherwise, the game moves on. When the game ends, *Player 1* is shown both the original picture and the ones with the outlined features to check that *Player 2* performed their task correctly. If any element has been improperly outlined or any question has been incorrectly answered, *Player 1* can provide their solution (i.e., answer and annotation). Such an action generates a conflict the researcher will resolve when the outcomes of the activity are provided.

Such an activity can be set up to have players mainly focus their questions on concrete features, abstract features or both. Moreover, such a gamified activity could be extended by applying the following changes, enhancing various steps of the activity:

- It would be possible to introduce a further step at the end of the activity where *Player 2* provides an additional picture of the same entity and outline the missing features on the new image.
- It would also be possible to introduce a further **Annotation Step** for *Player 1* at the end of the game to improve the reliability of the results, allowing the comparison of both players' annotation to identify inconsistencies in the provided outcomes.

## 4. Preliminary Evaluation

In this section, we report on a preliminary study on the effectiveness and impact of our approach. The experiments have been performed by selecting one entity category and by asking participants to interact over it. In particular, we picked “animals” as a category. We selected parrots and crocodiles as relevant representatives, and we collected a picture from Google Images for each of them. We purposely selected an image partially representing the crocodile (i.e., only its head was visible) and a complete one for the parrot. We engaged 30 people aged between 24 and 30, mostly (60%) employed in IT-related sectors. Most of them (75%) achieved an educational level superior or equal to a bachelor's degree. The participants were randomly organized into three groups:

- The “annotation” group (comprising 6 people), focusing on outlining features on images;
- The “gamified activity (concrete)” group (comprising 12 people) focusing their questions on concrete features (i.e., “R” and “NR” features);
- And the “gamified activity (generic)” group (comprising 12 people), where members were allowed to ask questions about any features.

Depending on such a division, each person was provided with a document describing their activity. The members of each of the gamified activity groups have been internally organized in pairs to carry out the game, thus generating 6 pairs per group. Each player was given one picture to play with. Players were asked to follow the same procedure described in 3.4, depending on their role and group. Each member of the pairs alternately played both roles. Overall, each group carried out 12 matches, (i.e., 6 matches per picture). Additionally, we asked people to keep track of each question and answer when playing as *Player 1*, and keep track of the suggestions provided when playing as *Player 2*. On the other hand, each of the 6 members of the “annotation” group was given two documents containing the chosen figures. They were appointed to describe the represented animal by providing a clear and short description of their features, its possible outline on the image, and its category.

## 5. Results and Discussion

### 5.1. Gamified Activity

Following the preliminary experiment, we discuss the outcomes and the feedback we collected, concerning the research questions we wanted to address.

With respect to **(Q1)**, aiming at assessing the role of the specific picture used in generating bias in the player describing the displayed object, we observed that (as expected) most of the concrete details reported by each “annotation” group member were represented in the picture, 73% for the crocodile and 97% for the parrot (Table 1A). Within the same group, we outlined a clear tendency to report “R” features first and forget about features not represented within the picture. Indeed, 50% of the participants provided no “NR” features for the partial image. These observations are aligned with our initial thoughts and expectations. When a person is asked to describe an entity, it mainly attains to the particular representation provided in the picture rather than the actual entity, even when it is well-known. Moreover, we observed a significant difference in the ratio between the amount of “NR” and concrete features collected for the partial picture among the different experiments. In particular, such proportion grew from 27% in the “Annotation” task to 34% in the “Gamified Activity (concrete).” Such a difference is even more emphasized in the “Gamified Activity (general)” experiment. We also identify a 50% increase in the total amount of “NR” features collected by the “Gamified Activity (concrete)” group with respect to the “annotation” one. Therefore, we may argue that creating a sharp separation of roles and hiding the picture from the gamified activity contributes to reducing the bias it induces.

**Table 1:** The table represents the average and the sample m.s.e. per participant for each feature type and for each picture.

**(A) “Annotation” Group**			
Picture	“R” Features	“NR” Features	“A” Features
Crocodile	3.6.7 ± 0.51	1.33 ± 1.63	1.5 ± 1.38
Parrot	5 ± 2	0.17 ± 0.48	2.17 ± 1.72
**(B) “Gamified Activity (concrete)” Group**			
Picture	“R” Features	“NR” Features	“A” Features
Crocodile	3.83 ± 1.94	2 ± 0.63	0.17 ± 0.41
Parrot	6 ± 0.89	0 ± 0	0.83 ± 0.41
**(C) “Gamified Activity (generic)” Group**			
Picture	“R” Features	“NR” Features	“A” Features
Crocodile	0.5 ± 0.55	1.17 ± 0.75	3.33 ± 0.81
Parrot	1.5 ± 0.55	0 ± 0	2.67 ± 1.51

*The table is organized depending on the groups described in Section 4*.

Regarding **(Q2)**, we argue that our methodology is able to identify more features w.r.t. a state of the art annotation method. Indeed, when the participants were asked to focus on concrete features (Table 1B), we observed a 20% increase in the number of “R” features for the picture of the parrot and a 33% increase in the number of “NR” features for the crocodile one, with respect to the features identified by the “annotation” group by using traditional methods. When analysing the outcomes of the “gamified activity (general)” group, we identified a clear tendency to ask questions about abstract features (e.g., “Is it carnivorous?,” “is it oviparous?,” “Does it live in the Jungle?,” “Is it able to speak?,” etc.) resulting in a 55% increase of abstract features collected with respect to the “Annotation” task (Table 1C). We believe such a behavior is strictly related to humans' capability to abstract concrete concepts and distinguish similar entities through peculiar and selective features, which (sometimes) are abstract. Questions on such selective features even played a fundamental role in the “Gamified Activity (concrete)” group, in which most people who had already collected a lot of concrete features, at the end of the process expressed the need to ask a few abstract questions to consolidate and finalize the identification of the animal. Furthermore, we believe that several descriptive dimensions, e.g., the selectivity of the features, and the category of the entity affect such behaviors.

We also collected some comments from the participants, whose feedback would lead to a significant improvement of the gamified activity. In particular, the following changes could be applied

- *Player 2* won't provide annotations for the collected features during the activity but only at the end. Such a change would smooth the flow of the activity, making it quicker and even more enjoyable for both players.
- At the end of the activity, both *Player 1* and *Player 2* will carry out the **Annotation Step**, improving the consistency of the results and the amount of data collected.
- At the end of the activity, both players will be shown the picture of the entity to further enrich the collection of the features they already identified by describing those they can derive from the entity's image.

In conclusion, we argue that our methodology extends gamified visual annotation and labeling methods, like the ones proposed in Runge et al. (2015) and Balayn et al. (2021a), mitigating the bias caused by pictures by hiding them, allowing an even more complete collection of features. Furthermore, our methodology can be easily extended by introducing further rules to shape and enhance its outcomes. Such an activity can be employed to collect data about what the model should know or should have learned about the entity. Such knowledge can be compared with the outcomes of other explainability methods to evaluate the difference between what the model knows and what it should know. Such a comparison can be carried out both for models learning from pictures of the entity - by comparing the heat maps derived from the model and the annotated “R” (and optionally “NR”) features—and textual descriptions of the entity—comparing the outcomes of saliency-based analyses and the collected features. Moreover, the collected knowledge could be further combined to enhance the outcomes of non-textual, local explainability methods or improve the textual description of textual ones. In particular, non-abstract features annotated by the crowd would be useful to describe pictures in which the same feature is detected by other methods (e.g., heat maps, etc.), while abstract details would be useful to complete textual descriptions, making them more human-understandable and human-like.

### 5.2. Framework

We argue that our framework would facilitate and structure the exchange of knowledge between the research community and the crowd, leading to an overall improvement of the content provided and the level of understanding of the engaged users. Moreover, the presented crowdsourcing framework engages the users on a different level with respect to other platforms mainly based on extrinsic rewards. In particular, user education would improve users' awareness of what kind of knowledge an AI system needs, learns, and produce, enhancing their efficiency and shaping their mindset. Such a statement would also be amplified when a long-term engagement of the users is achieved.

We are aware of the limitations implied by our framework, namely the initial engagement gap, the necessity of keeping the users and the researchers engaged, and the high level of flexibility required to cover all the explainability-related aspects. Gamification will be helpful to compensate for the first two aspects, while the last one will be covered through an accurate design of the proposed activities. In particular, the design will include both extrinsic and intrinsic design elements to account for both the initial and long-term engagement, respectively. In particular, users' side extrinsic design elements will consist of points, activity leaderboards, achievements (i.e., status as a reward), etc. Intrinsic design elements will be mainly associated with the education aspect as it is strictly related to one of the three innate psychological needs (Ryan and Deci, 2000), namely Competence (i.e., people are wishful to learn new skills and mastery tasks). On the other hand, we expect researchers to be engaged as they trade their scientific knowledge for data for their research. Moreover, developing a cooperative framework is challenging, especially when users and researchers must be engaged. We plan to engage users using renowned crowdsourcing platforms for testing purposes, while the initial engagement on the final release will be performed through the university and researchers' network.

## 6. Conclusions and Future Works

We presented the preliminary design of a crowdsourcing framework to create a cooperative cycle in which the crowd is taught about explainability-related topics and provides valuable content to AI practitioners. Gamification is applied to empower engagement and drive user behavior. The design and the preliminary evaluation of a gamified data collection activity is also provided. We argue that our research would improve the quality of the data collected to evaluate and enhance the explainability of black-box models. Future work will involve the improving of the design of both the presented activity—following the discussed changes—and the framework. We plan to execute further experiments to generalize the results on effectiveness and efficiency of our method, and to release an opensource crowdsourcing platform, which may be adopted by the broader research community.

## Data Availability Statement

The original contributions presented in the study are included in the article/supplementary material, further inquiries can be directed to the corresponding author/s.

## Author Contributions

All authors listed have made a substantial, direct, and intellectual contribution to the work and approved it for publication.

## Conflict of Interest

The authors declare that the research was conducted in the absence of any commercial or financial relationships that could be construed as a potential conflict of interest.

## Publisher's Note

All claims expressed in this article are solely those of the authors and do not necessarily represent those of their affiliated organizations, or those of the publisher, the editors and the reviewers. Any product that may be evaluated in this article, or claim that may be made by its manufacturer, is not guaranteed or endorsed by the publisher.
